# The histone deacetylase inhibitor valproic acid inhibits NKG2D expression in natural killer cells through suppression of STAT3 and HDAC3

**DOI:** 10.1038/srep45266

**Published:** 2017-03-24

**Authors:** Lulu Ni, Lixin Wang, Chao Yao, Zhongya Ni, Fei Liu, Chenyuan Gong, Xiaowen Zhu, Xuewei Yan, Stephanie S. Watowich, Dean A. Lee, Shiguo Zhu

**Affiliations:** 1 Laboratory of Integrative Medicine, School of Basic Medical Sciences, Shanghai University of Traditional Chinese Medicine, 1200 CaiLun Rd., Shanghai 201203, P. R. China; 2 Department of Immunology and Pathogenic Biology, School of Basic Medical Sciences, Shanghai University of Traditional Chinese Medicine, 1200 CaiLun Rd., Shanghai 201203, P. R. China; 3 Department of Immunology, The University of Texas MD Anderson Cancer Center, Houston, TX, 77030, USA; 4 Pediatrics, Nationwide Children’s Hospital, 700 Children’s Drive, WA4023, Columbus, OH, 43205, USA.

## Abstract

NKG2D is a major activating receptor of NK cells and plays a critical role in tumor immunosurveillance. NKG2D expression in NK cells is inhibited by the histone deacetylase (HDAC) inhibitor valproic acid (VPA) and enhanced by the narrow-spectrum HDAC inhibitor entinostat. We previously demonstrated that entinostat enhanced NKG2D transcription by increasing acetylation of Histones H3 and H4. However, the mechanism by which VPA reduces NKG2D expression in NK cells is not known. We have also shown that NKG2D transcription is regulated by STAT3 phosphorylation. In this study, we investigated regulation of NKG2D expression in NK cells by VPA and entinostat by assessing protein expression, phosphorylation, and interaction of HDACs and STAT3. We find that VPA selectively inhibits STAT3 tyrosine705 phosphorylation, but entinostat does not. STAT3 complexes with HDAC3, and HDAC3 inhibition represses STAT3 phosphorylation and therefore NKG2D expression. NK cells from STAT3 wild-type mice downregulate NKG2D in response to VPA, but not NK cells from STAT3 knockout mice. These results show that VPA is a potent inhibitor of STAT3 phosphorylation and demonstrate that histone acetylation and STAT3 tyrosine705 phosphorylation cooperate in regulating NKG2D expression in NK cells.

Natural Killer (NK) cells are large granular lymphocytes that play a critical role in the host defense against viral infection and cancers. NK cell-mediated lysis of target cells is determined by the balance of signaling between activating and inhibitory receptors on NK cells^1^. NKG2D is the major activating receptor of NK cells and plays a crucial role in tumor immunosurveillance^2^. In humans, NKG2D recognizes and binds to MIC A/B (MHC class I–related chains A and B) and ULBPs (UL16-binding proteins) on target cells and exerts an activating signal to promote NK cell cytotoxicity. NKG2D expression can be enhanced by the cytokines IL-2, -12, -15, and -21^3^, and is suppressed by the inflammatory factors TGF-β, prostaglandin E2 (PGE2) and L-kynurenine, a tryptophan catabolite generated by indoleamine-2,3-dioxygenase (IDO)^4^,^5^,^6^. Enhancement of NKG2D expression on NK cells or of its ligands on cancer cells is a potential strategy for improving cancer immunotherapy.

Histone deacetylases (HDACs) are enzymes catalyzing the removal of acetyl groups, leading to chromatin condensation and transcriptional repression^7^. HDACs are involved in cell proliferation, apoptosis, differentiation, migration, and metastases in cancers^8^. Therefore, HDACs have become promising targets for cancer treatment, and several classes of HDAC inhibitors have been developed and have been tested in clinical trials^9^. HDAC inhibitors (HDACi) such as valproic acid (VPA) and entinostat have been shown to sensitize NK cell-mediated killing by upregulating expression of NKG2D ligands MICA/B or ULBP1on cancer cells^10^,^11^,^12^,^13^,^14^,^15^, suggesting that HDACi might have promising applications in cancer immunotherapy, especially in combination with adoptive NK cell therapy.

However, the HDACi VPA has been found to impair NK cell cytolytic activity by decreasing expression of NKG2D on NK cells^16^,^17^. This is in contrast to our finding that the HDACi entinostat enhances NK cell cytolytic activity by increasing expression of NKG2D on NK cells^15^. We showed that entinostat enhances the expression of NKG2D in NK cells through inhibiting HDAC1 and HDAC2, thereby increasing acetylation of histones H3 and H4 which result in increased NKG2D transcription. VPA also inhibits HDAC1 and HDAC2, so we questioned why VPA reduces the expression of NKG2D in NK cells rather than enhancing it. Understanding the mechanism of VPA-induced suppression of NKG2D expression in NK cells would be valuable for developing HDAC as targeted cancer drugs for NK cell immune modulation.

To elucidate the mechanism by which VPA down-regulates NKG2D expression in NK cells, we assessed the expression and regulation of NKG2D in NK cells after VPA treatment by analyzing protein expression, phophorylation and interaction, using entinostat as a control. We found that HDAC3 was able to be complexed with STAT3; VPA inhibited HDAC3 and then resulted in selectively inhibition of STAT3 tyrosine705 phosphorylation, and subsequent down-regulation of NKG2D expression in NK cells. This finding indicates that HDAC3 and STAT3 signaling cooperate in the regulation of NKG2D expression in NK cells, and provides valuable insight for new drug development and clinical applications of HDACi in cancer treatment.

## Results

### HDACi VPA reduces but entinostat enhances NKG2D expression in NK cells

**Figure 1:**
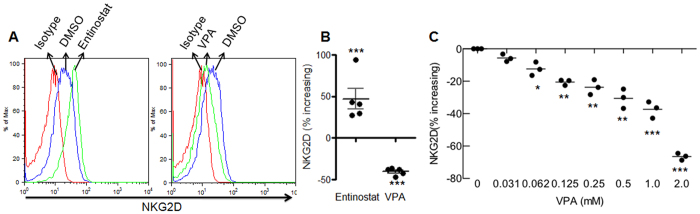
Effect of HDAC inhibitors on NKG2D expression after 24 hrs treatment. NK cells were treated with 0.1 μM of entinostat or 0.5 mM of VPA for 24 hrs, NKG2D expression was accessed by flow cytometry. (**A**) Representative of 5 independent experiments. (**B**) Percentage increasing of NKG2D expression was calculated by the formula: (NKG2D Mean in treated NK cells - NKG2D Mean in untreated NK cells)/NKG2D Mean in untreated NK cells *100. The data were pooled from 5 donors. (**C**) Effect of VPA concentration on NKG2D expression. *p < 0.05; **p < 0.01; ***p < 0.001.

To further define the regulation of NKG2D in NK cells by HDACi, we treated primary human NK cells with the wide-spectrum HDACi VPA and the narrow-spectrum HDACi entinostat. We found that VPA ≥ 1 mM and entinostat ≥1 μM significantly impaired NK cell viability, but VPA ≤ 0.5 mM and entinostat ≤0.1 μM did not (Suppl. Fig. 1A,B). Since these values also fall within the published *in vitro* effective-dose concentrations for these two inhibitors^18^,^19^, we treated NK cells with 0.5 mM VPA or 0.1 μM entinostat and found that VPA reduced, but entinostat enhanced NKG2D expression (Fig. 1A). We then analyzed the increasing percentage ratio of NKG2D expression with VPA or entinostat compared to untreated control, we found VPA significantly decreased but entinostat significantly increased NKG2D expression (Fig. 1B). To further determine the effect of VPA, we used varying concentrations of VPA to treat NK cells and found that VPA down-regulated NKG2D expression in NK cells in a dose-dependent manner (Fig. 1C). These results showed that the narrow-spectrum HDACi entinostat and the wide-spectrum HDACi VPA exert opposite effects on NKG2D expression in NK cells.

### VPA impairs NK cell degranulation and cytotoxicity

**Figure 2:**
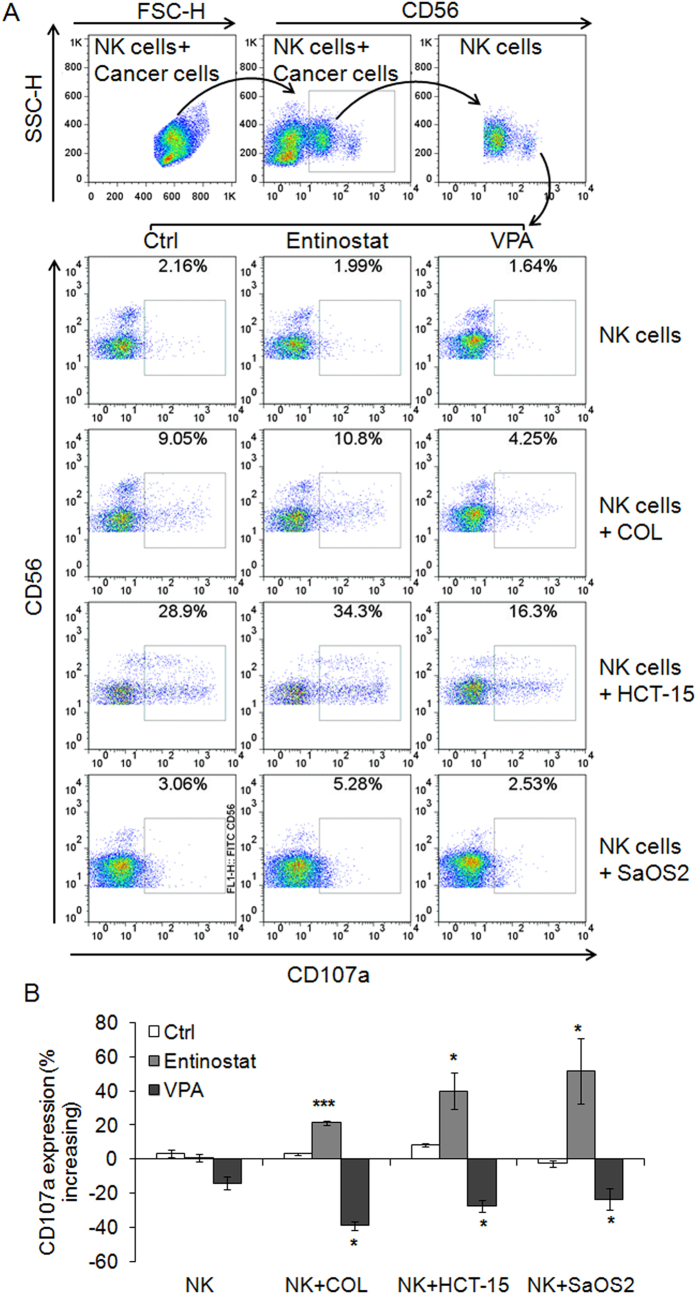
VPA impairs but entinostat enhances NK cell degranulation. Primary human NK cells were treated with 0.1 μM of entinostat and 0.1 mM of VPA for 24 hrs, respectively; and then co-incubated with COL, HCT-15 and SaOS2 cells (E:T = 4:1) for 4 hrs. NK cell degranulation was evaluated by flow cytometry for CD107a expression. (**A**) Representative dot plot; (**B**) Percentage increasing of CD107a expression was calculated by the formula: (percentage CD107a in treated NK cells - percentage CD107a in untreated NK cells)/percentage CD107a in untreated NK cells *100. The data are expressed as mean ± SEM of three independent experiments. *p < 0.05; **p < 0.01; ***p < 0.001.

Since VPA down-regulated NKG2D expression in NK cells, we next determined whether VPA impaired NK cell antitumor activity. CD107α expression on the surface of NK cells is a sensitive marker of NK cell functional activity and correlates with NK cell degranulation and cytotoxicity^20^. We used 0.1 μM entinostat and 0.1 mM of VPA to treat primary human NK cells for 24 h, and then co-incubated NK cells with COL, HCT-15 and SaOS2 cells for 4 h, respectively. We found that entinostat enhanced CD107 surface expression on NK cells, but VPA clearly reduced it (Fig. 2). We also determined the effect of VPA on NK cell cytoxicity by the calcein release assay and found that VPA significantly inhibited NK cell cytotoxicity in a dose-dependent manner (Suppl. Fig. 2). These results showed that VPA has a significant inhibitory effect on NK cell degranualtion and cytotoxicity.

### VPA inhibits STAT3 Tyrosine705 phosphorylation

**Figure 3:**
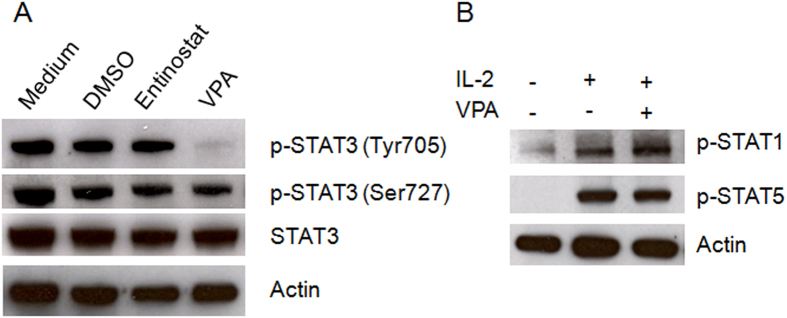
VPA inactivates STAT3 in NK cells. (**A**) Expression and phosphorylation of STAT3 in purified primary NK cells with or without treatment with 1.0 μM entinostat or 0.1 mM VPA. (**B**) Phosphorylation of STAT1 and STAT5 in purified primary NK cells with or without treatment with 0.1 mM VPA and IL-2. Similar results were obtained in three independent experiments.

In our previous study, we found that signal transducer and activator of transcription-3 (STAT3) phosphorylation is critical for the transcriptional expression of NKG2D in NK cells^21^; this led us to determine the effect of VPA on STAT3 phosphorylation. We treated primary human NK cells for 24 h with 1 μM entinostat or 0.1 mM VPA and then assessed STAT3 phosphorylation. We found that VPA inhibited STAT3 tyrosine705 phosphorylation, and had no effect on total STAT3 protein expression and serine727 phosphorylation. Entinostat had no effect on STAT3 protein expression or phosphorylation (Fig. 3A). To further determine the selective action of VPA on STAT3 inhibition, we measured the phosphorylation of STAT1 and STAT5 in NK cells after treatment with VPA and found that VPA did not affect STAT1 and STAT5 phosphorylation (Fig. 3B). These results indicated that VPA selectively inhibits STAT3 tyrosine705 phosphorylation, and suggested that down-regulation of NKG2D expression by VPA in NK cells might be mediated through inactivation of STAT3.

### VPA-induced down-regulation of NKG2D expression is abrogated by STAT3 deletion

**Figure 4:**
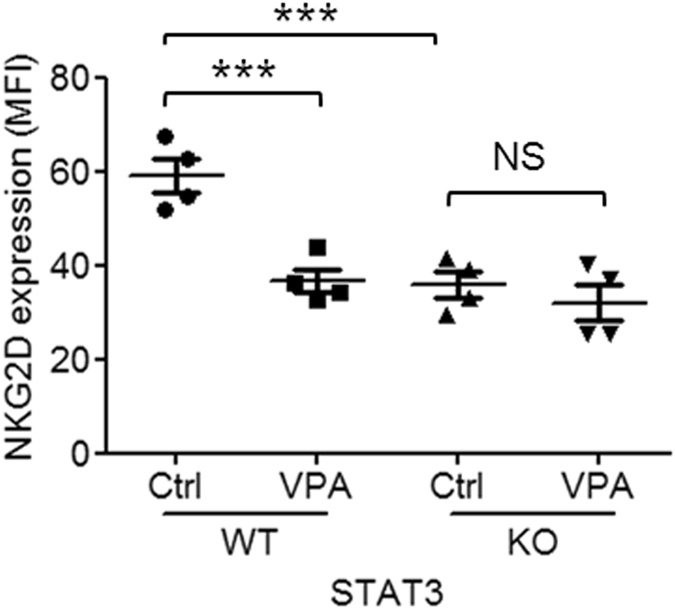
NKG2D expression on NK cells from STAT3 wild-type and knockout mice. Murine NK cells were isolated from spleens of STAT3 wild-type and knockout mice, and then treated with/without 0.1 mM VPA for 24 hrs. NKG2D surface expression on CD3-NKp46+ cells was analyzed by flow cytometry. The data was pooled from 4 mice. *p < 0.05; **p < 0.01; ***p < 0.001; NS, non-significant.

As the above results showed that VPA inhibited phophorylation of STAT3 tyrosine705 in NK cells, we next determined whether STAT3 is necessary for VPA-inducted down-regulation of NKG2D. We assessed NKG2D expression in NK cells from mice with conditional deletion of floxed STAT3 limited to hematopoietic cells through Tie2 activation of Cre. We found that NKG2D expression was significantly lower in NK cells from STAT3 KO mice compared to WT mice. VPA could significantly down-regulate NKG2D expression in NK cells from STAT3 WT mice, but did not decrease NKG2D expression in NK cells from STAT3 KO mice (Fig. 4). These results demonstrate that STAT3 is required for VPA-induced down-regulation of NKG2D expression in NK cells.

### STAT3 interacts with HDAC3

**Figure 5:**
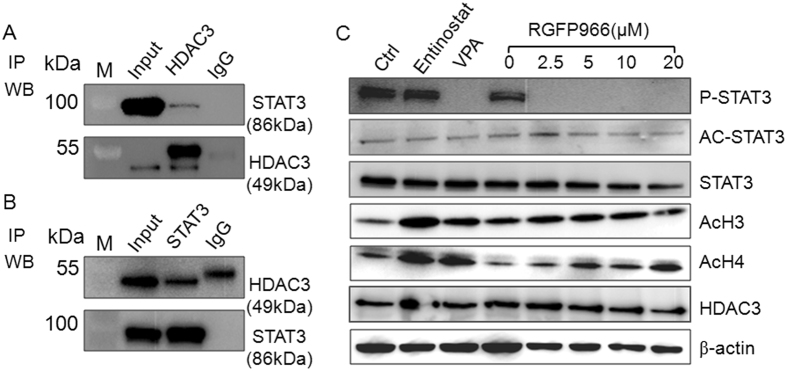
STAT3 interacts with HDAC3 in NK cells. (**A**) Proteins were immunoprecipitated from NK cell lysate by HDAC3 monoclonal antibody and isotype IgG, and then detected by STAT3 and HDAC3 antibodies using two separate blots with the same sample to avoid the HDAC3 (49 kDa) to be overlapped with the antibody heavy IgG (55 kDa). (**B**) Proteins were immunoprecipitated from NK cell lysate by STAT3 monoclonal antibody and isotype IgG, and then detected by HDAC3 and STAT3 antibodies using two separate blots with the same sample. (**C**) NK cells were treated with Entinostat, VPA and RGFP966. Phosphorylation, acetylation and total protein of STAT3 and acetylated histone H3/H4 were detected by western-blot. Similar results were obtained in three independent experiments.

Having demonstrated that VPA is an HDAC inhibitor that also inhibits STAT3 phosphorylation, we questioned whether HDAC deacetylase activity is required for STAT3 phosphorylation. VPA mainly inhibits the class I (HDAC1, 2, 3 and 8) and class IIa (HDAC4, 5, 7 and 9) HDACs^9^, and inhibits class I more efficiently than class II enzyme HDACs 5 and 6^19^. By a prelimary screening (data not shown), we found that STAT3 could be pull-downed by HDAC3, which suggested HDAC3 might be complexed with STAT3. To further confirm this issue, we first used HDAC3 antibody to pull-down and then immunoblotted with STAT3, we found HDAC3 pull-down actually had a clear STAT3 band (Fig. 5A). We then immunoprecipitated STAT3 followed by immunoblotting with HDAC3 antibody. We found that STAT3 and HDAC3 clearly co-precipitated compared to isotype IgG (Fig. 5B). Since STAT3 could bind with HDAC3, this suggested that HDAC3 might play a role in STAT3 phosphorylation. To assess this functional interaction we treated human NK cells with the HDAC3-specific inhibitor RGFP966^22^. We found that RGFP966 inhibited the phosphorylation of STAT3 (Fig. 5C), but neither VPA nor RGFP966 affected the acetylation of STAT3. These results show that STAT3 complexes with HDAC3 in NK cells, and HDAC3 is required for STAT3 phosphorylation.

### HDAC3 inhibition down-regulates NKG2D expression

**Figure 6:**
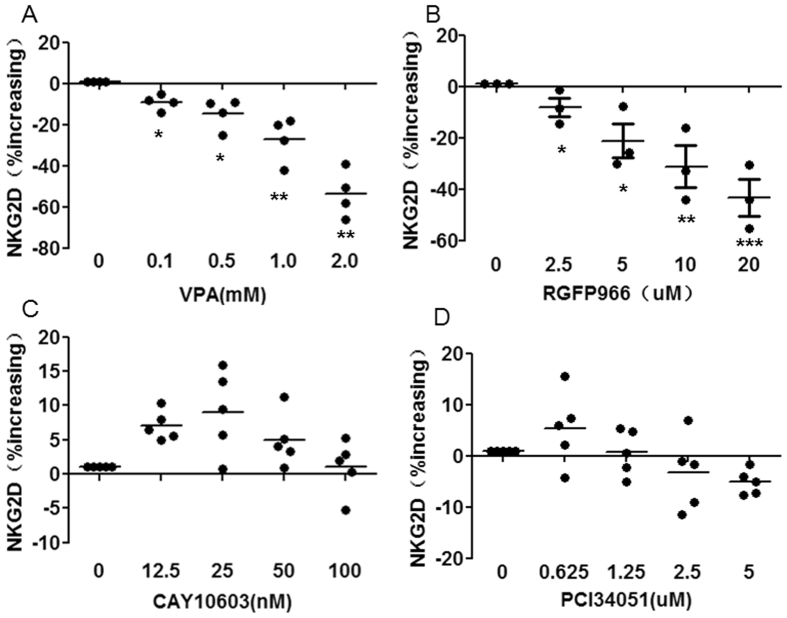
NKG2D expression on NK cells after treatment with VPA, RGFP966, CAY10603 and PCI34051. NK cells were treated with indicated concentrations of VPA, RGFP966, CAY10603 and PCI34051 for 24 hrs, and then NKG2D surface expression was analyzed by flow cytometry. The data are pooled from 3–5 donors. *p < 0.05; **p < 0.01; ***p < 0.001.

Having determined that HDAC3 was required for STAT3 phosphorylation and having previously shown that STAT3 phosphorylation is required for NKG2D expression in NK cells, we next determined the effect of HDAC3 on NKG2D expression. We used the HDAC3-specific inhibitor RGFP966^22^, HDAC6-specific inhibitor CAY10603^23^, and HDAC8-specific inhibitor PCI34051^24^ to treat expanded NK cells. We found that VPA ≤ 0.5 mM, RGFP966 ≤ 20 μM, CAY10603 ≤ 50 nM and PCI34051 ≤ 0.625 μM did not impair NK cell viability at 24 h (Suppl. Fig. 3). The HDAC3 inhibitor RGFP966 caused down-regulation of NKG2D expression on NK cells in a dose-dependent manner similar to VPA, but the HDAC6 inhibitor CAY10603 and the HDAC8 inhibitor PCI34051 did not decrease NKG2D expression (Fig. 6). These results show that HDAC3 inhibition down-regulates NKG2D expression in NK cells and indicates that HDAC3 is required for NKG2D expression.

## Discussion

In this study, we sought to identify the mechanism by which VPA decreases expression of NKG2D in NK cells. We found that VPA reduced but entinostat enhanced expression of NKG2D and NK cell degranulation. We also found that VPA inhibited STAT3 tyrosine705 phosphorylation but entinostat did not, suggesting that STAT3 phosphorylation was critical for NKG2D expression in NK cells. We then demonstrated that STAT3 interacts with HDAC3, and that HDAC3 inhibition represses STAT3 tyrosine705 phosphorylation - and therefore NKG2D expression - in NK cells. Furthermore, VPA-induced down-regulation of NKG2D is abrogated by STAT3 deletion. These results clearly show that VPA-mediated inhibition of NKG2D expression in NK cells is dependent on both STAT3 and HDAC3.

STAT3 promotes tumorigenesis as an oncogene by regulating the expression of various target genes that mediate cell cycle, survival, proliferation, apoptosis, invasion, angiogenesis, and metastasis^25^. STAT3 also promotes tumor immune escape by governing multiple immunosuppressive mechanisms including macrophage polarization to the M2 phenotype, inhibition of DC development, and accumulation of immunosuppressive cells such as Tregs, Th17 cells, and MDSCs^26^. Therefore, a strong interest has developed in STAT3 as a potent therapeutic target for cancer treatment. A number of STAT3 inhibitors have been developed and tested in clinical trials, but so far no STAT3-specific inhibitor has been approved for clinical use^27^.

In our previous study, we found that STAT3 is required for NKG2D expression and cytotoxicity in human NK cells^21^. In the current study, we demonstrated that VPA inhibits phosphorylation of STAT3 and thereby reduces expression of NKG2D in NK cells from wild-type mice, but not in NK cells from STAT3 knockout mice. These results indicate that STAT3 is required for NK cell-mediated immunosurveillance through modulation of NKG2D.

In the current study, we found that STAT3 interacts with HDAC3, and HDAC3 inhibition by VPA or the HDAC3-specific inhibitor RGFP669 reduced expression of NKG2D in NK cells. This shows that HDAC3 regulates NKG2D expression in NK cells, confirming the regulation of STAT3 by HDAC3 that was recently demonstrated in diffuse large B cell lymphoma^28^. In lymphoma, inhibition of HDAC activity by panobinostat (LBH589) increased STAT3 Lys685 acetylation^28^, but in our study we found that entinostat, VPA and RGFP669 did not affect STAT3 acetylation. This may be due to the difference of B cells and NK cells, or more broadly of cancer cells and normal cells.

**Figure 7:**
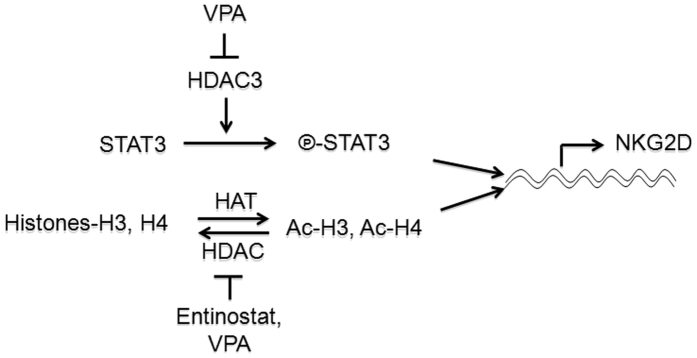
Model of transcriptional regulation of NKG2D by the phosphorylation of STAT3 and acetylation of Histones-H3 and H4 in NK cells. HDAC3 is required for STAT3 phospharylation which determines NKG2D transcript in NK cells. VPA inhibits HDAC3 and results in suppression of STAT3 phosphorylation and then reduction of NKG2D expression in NK cells. Histones-H3 and H4 can be acetylated by HAT, and acetylated-H3 and H4 can be deacetylated by HDAC. Entinostat preferentially inhibits HDAC1 and is less active against HDAC3 resulting in enhanced acetylation of H3 and H4 and then increased binding of acetylated H3 and H4 (Ac-H3, Ac-H4) to the promoter of NKG2D, promoting NKG2D transcription.

We showed that NKG2D expression in NK cells may be regulated by the phosphorylation of STAT3 in this study, and by the acetylation of Histones-H3 and H4 in our previous study^15^ (Fig. 7). Entinostat preferentially inhibits HDAC1, is less active against HDAC3, and only marginally inhibits HDAC8^29^, in contrast to VPA inhibits the class I(HDAC1, 2, 3 and 8) and class IIa (HDAC4, 5, 7 and 9) HDACs^9^. However, VPA decreases but entinostat enhances NKG2D expression, which suggests that regulation of NKG2D expression in NK cells is more dependent on HDAC3-STAT3 signaling than HDAC1-Ac-H3/4 signaling.

We assessed the expression and regulation of NKG2D in NK cells by HDAC inhibitors VPA and entinostat, Taken together, we found that VPA inhibits HDAC3 resulting in selective inhibition of phosphorylation of STAT3 tryrosine705, which down-regulates NKG2D expression in NK cells. These findings show that HDAC3-STAT3 signaling regulates NKG2D expression in NK cells, suggesting that the development of new cancer drugs should avoid impairing this signal pathway.

## Methods

### Reagents

Murine monoclonal antibodies against human CD3, CD56, CD107a, and NKG2D, antibodies against murine CD3, NKp46, and NKG2D, and isotype control monoclonal antibodies were obtained from BioLegend Inc. (San Diego, CA). The recombinant human IL-2 was obtained from PeproTech (Rehovot, Israel). Entinostat, VPA, PCI24781, RGFP966, CAY10603 and PCI34051were obtained from Selleck (Texas, USA). Antibodies against human pSTAT1 (Tyr 701), STAT3, pSTAT3 (Tyr 705), p-STAT3 (Ser727), pSTAT5 (Tyr 694), HDAC1–9 and β-actin were obtained from Santa Cruz Biotechnology, Inc. (Santa Cruz, CA). Cell Counting Kit (CCK8) was purchased from YESAN (Shanghai, China). Calcein-AM was purchased from Sigma-Aldrich (St. Louis, MO).

### Cell culture

COL (neuroblastoma) and HCT-15 (colon carcinoma) were obtained as previously described and cultured in Dulbecco’s modified Eagle’s medium (DMEM) supplemented with 10% fetal bovine serum and 1% penicillin and streptomycin. SaOS2 cells from ATCC were cultured in RPMI1640 medium (Gibco) supplemented with 10% of fetal calf serum (Gibco), 1% of penicillin-streptomycin, and 2 mM of L-Glutamine in 5% CO_2_ at 37 °C.

### NK cell purification

Human peripheral blood mononuclear cells (PBMC) were obtained from the Shanghai Blood Center under a research protocol approved by the Department of Shanghai Blood Administration. Human NK cells were purified from PBMC using the RosetteSep Human NK Cell Enrichment Cocktail (StemCell Technologies, Vancouver, BC, Canada) as described previously^30^. Murine splenocytes were obtained from wild-type (WT) mice or mice in which floxed STAT3 was deleted in hematopoietic cells by Cre expression under the Tie2 promoter^31^. Murine NK cells were isolated from splenocytes using the EasySep Mouse NK Cell Enrichment Kit (Stemcell Technologies Vancouver, BC, Canada). All experiments involving animals were conducted in accordance with Institutional Animal Care and Use Committee approval at University of Texas MD Anderson Cancer Center.

### Human NK cell expansion

Human NK cells were expanded from PBMC *in vitro* by weekly stimulation with mbIL-21-CD137L-K562 cells in the presence of 100 U/mL of IL-2 for 2–3 weeks as described previously^32^. PBMCs were either freshly used or frozen in 10% of DMSO containing FBS. For frozen PBMCs, PBMCs were thawed 1 day prior to their cultivation in RPMI 1640 medium supplemented with 10% of FBS, 1% of penicillin-streptomycin, 2 mM of L-Glutamine and 200 U/ml of IL-2 in 5% CO2 at 37 °C.

### Cell viability

To determine the effect of VPA, PCI24781, RGFP966, CAY10603 and PCI34051 on the viability of NK cells, CCK8 assay was performed. 1 × 10^4^ of NK cells were seeded per well in 96-well plates. VPA, PCI24781, RGFP966, CAY10603 and PCI34051 were added to the indicated concentration. 24 hrs later, 10 μL of CCK8 was added per well. Plates were incubated for 1 hr and then read at 450 nm using Synergy 2 Multi-Mode Microplate Reader (BioTek Instrument, Int., Winooski, VT). Independent experiments at least were performed three times.

### Degranulation Assay

To evaluate the effect of VPA on NK cell degranulation, CD107α surface expression on NK cells was analyzed. NK cells were treated with 0.1 mM of VPA or 0.1 μM of entinostat for 24 hrs, and then cocultured with COL, HCT-15 or SaOS2 cells at a 4:1 ratio with anti–human CD107α or isotype control antibodies present during the coculture period. After 4 hrs co-incubation, cells were stained with anti-human CD56, and NK cell degranulation (CD56+CD107α+) was assessed by flow cytometry.

### Flow Cytometry

Cells were exposed to appropriate fluorescence-conjugated antibodies for 30 min at 4 °C in the dark, and then washed and resuspended in 1% FBS containing PBS. Data was acquired by a BD Accuri C6 (BD Biosciences), and analyzed using FlowJo software (Ashland, OR).

### Calcein Release Assay

NK cell tumoricidal activity was determined by the calcein release assay as described previously^33^. Briefly, primary NK cells were incubated with VPA or DMSO for 24 hrs. Target cells were labeled with 2 μg/mL of calcein-AM for 1 hr at 37 °C with occasional shaking. Effector cells and target cells were cocultured at 40:1 ratio and incubated at 37 °C for 4 hrs. After incubation, 100 μL of the supernatant was harvested and transferred to a new plate. Absorbance at 570 nm was determined using a Synergy 2 Multi-Mode Microplate Reader (BioTekInstrument, Int., Winooski, VT). Percent lysis was calculated according to the formula [(experimental release-spontaneous release)/(maximum release − spontaneous release)] × 100.

### Western Blot Analysis

NK cells were incubated as indicated with entinostat (1.0 μM), VPA (0.1 mM), RGFP966 (0, 2.5, 5.0, 10 and 20 uM) for 24 hrs and then were lysed with 50 mM Tris-Cl (pH 6.8), 100 mM dithiothreitol, 2% SDS, and 10% glycerol. Samples were quantitated using BCA protein reagent assay kit (Yeasen, Shanghai) and analyzed by 8~10% of SDS-PAGE, followed by immunoblotting using Enhanced Chemiluminescence Substrate (MerckMillipore, USA) according to the manufacturer’s instructions. Bands were visualized using a chemiluminescent detection system (ProteinSimple, USA).

### Co-Immunoprecipitation

NK cells were lysed at room temperature for 30 min in 1 ml lysis buffer (25 mM Hepes (pH 7.4), 5 mM EDTA, 50 mM NaCl, 10% glycerol, 1% Triton X-100, 50 mM NaF, 30 mM sodium pyrophosphate and protease inhibitors (1 μg/ml each of aprotinin, leupeptin and pepstatin A, and 1 mM phenyl-methylsulfonyl fluoride)). The lysates were cleared by centrifugation at 12,000 g for10 min and protein content of the supernatant was evaluated using a BCA protein assay reagent kit (Yeasen, Shanghai). For co-immunoprecipitation, 500–1000 μg protein/ml was mixed with either 4 μg anti-HDAC3 antibody or 4 μg anti-STAT3 antibody (Santa Cruz Biotechnology) for 3 hrs. The antibody–antigen complexes were precipitated by protein G-linked Sepharose (Pharmacia) for 30 min and the beads were washed twice with lysis buffer. The denatured co-immunoprecipitation products were subjected to SDS-PAGE analysis, followed by immunoblotting using Enhanced Chemiluminescence Substrate (MerckMillipore, USA) according to the manufacturer’s instructions. Bands were visualized using a chemiluminescent detection system (ProteinSimple, USA).

### Statistical analysis

Results are expressed as the mean ± standard error of the mean (S.E.M). One way ANOVA and independent samples t-test were used to measure statistical significance between the mean in all experiments. P-values of less than 0.05 were considered significant.

## Additional Information

**How to cite this article**: Ni, L. *et al*. The histone deacetylase inhibitor valproic acid inhibits NKG2D expression in natural killer cells through suppression of STAT3 and HDAC3. *Sci. Rep.* **7**, 45266; doi: 10.1038/srep45266 (2017).

**Publisher's note:** Springer Nature remains neutral with regard to jurisdictional claims in published maps and institutional affiliations.

## Supplementary Material

Supplementary Data
