# Supplementary material for: The histone deacetylase inhibitor valproic acid inhibits NKG2D expression in natural killer cells through suppression of STAT3 and HDAC3
Source: Sci Rep. 2017 Mar 24;7:45266. doi: 10.1038/srep45266 (PMC5364405; doi:10.1038/srep45266)
Supplement: Supplementary Data [file srep45266-s1.pdf]

The histone deacetylase inhibitor valproic acid inhibits NKG2D expression in natural killer cells through suppression of STAT3 and HDAC3

Lulu Ni[1]*, Lixin Wang*[2], Chao Yao[1],Zhongya Ni[1], Fei Liu[1], Chenyuan Gong[1], Xiaowen Zhu[1], Xuewei Yan[1], Stephanie S. Watowich[3], Dean A. Lee[4#], Shiguo Zhu[1, 2 #]

[1]Laboratory of Integrative Medicine, School of Basic Medical Sciences, Shanghai University of Traditional Chinese Medicine, 1200 CaiLun Rd. Shanghai 201203, P.R. China.

[2]Department of Immunology and Pathogenic Biology, School of Basic Medical Sciences, Shanghai University of Traditional Chinese Medicine, 1200 CaiLun Rd. Shanghai 201203, P.R. China.

[3]Department of Immunology, The University of Texas MD Anderson Cancer Center, Houston, TX 77030.

[4]Pediatrics, Nationwide Children's Hospital, 700 Children's Drive, WA4023, Columbus, OH 43205.

*These authors contributed equally to this work.

#Correspondence should be addressed to shiguozhu, zhushiguo@shutcm.edu.cn and Dean A. Lee, Dean.Lee@nationwidechildrens.org .

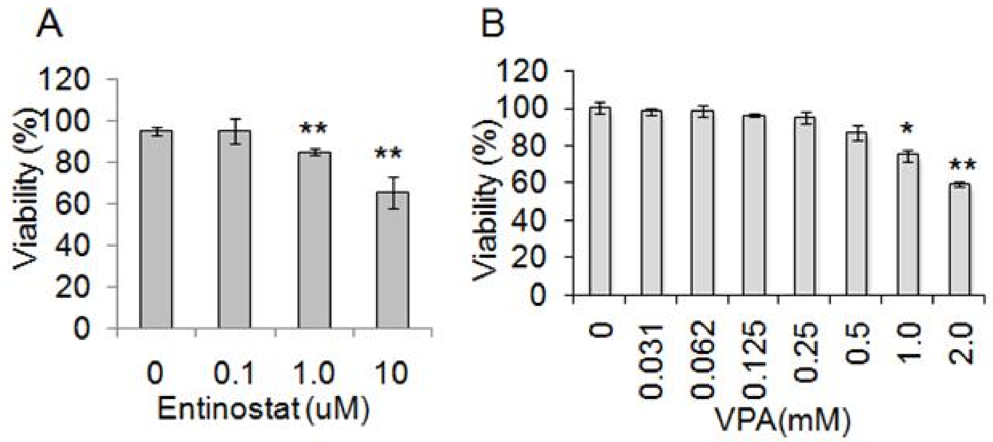

Suppl. Fig 1. Influence of Entinostat and VPA on the viability of primary NK cells. A, entinostat; B, VPA. The data are expressed as mean ± S.E.M. of three independent experiments. *, P<0.05; **, P<0.01; ***, <0.001.

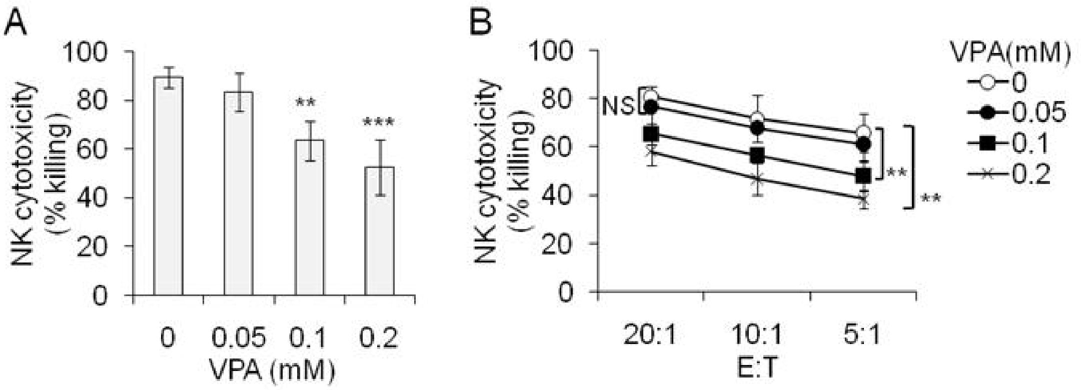

Suppl. Fig 2. Influence of VPA on NK cell cytotoxicity. Different concentrations (0, 0.05, 0.1 and 0.2mM) of VPA were used to treat primary NK cells for 24 hrs. NK cell cytotoxicity was evaluated by calcein release assay. A, NK cell cytotoxicity at an E:T ratio of 40:1, the data are expressed as mean ± S.E.M. of three independent experiments.; B, NK cell cytotoxicity at different of E:T ratios, the data are representative of three independent experiments. *, P<0.05; **, P<0.01; ***, <0.001,

NS, non-significant.

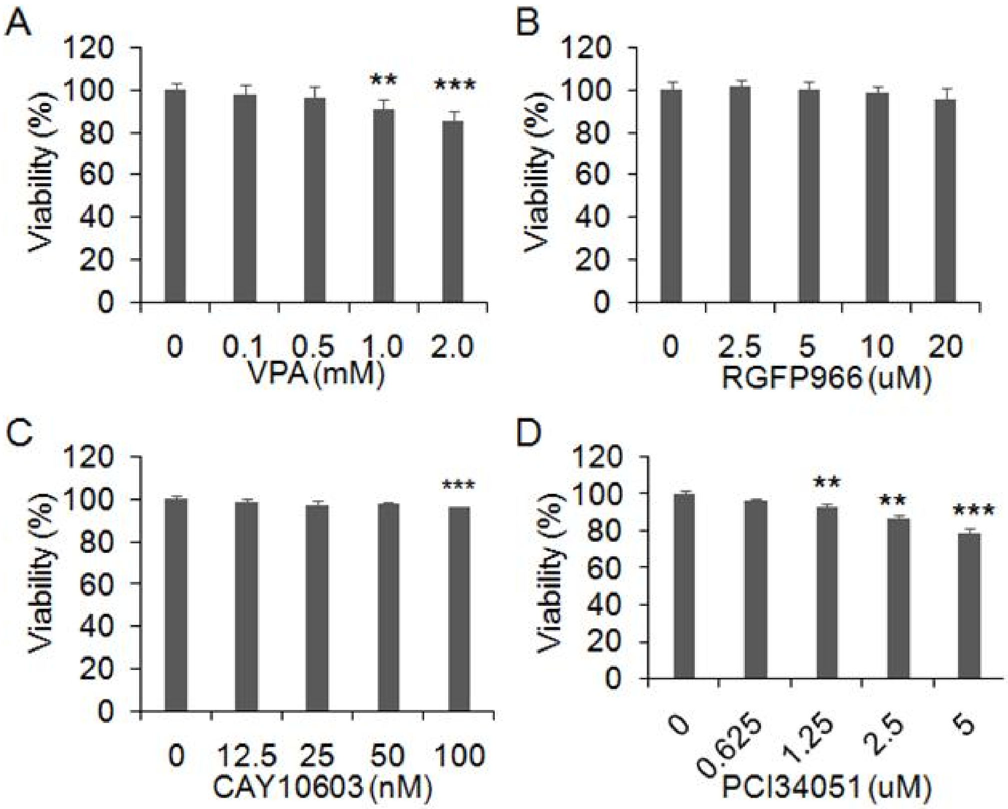

Suppl. Fig 3. Influence of VPA, RGFP966, CAY10603 and PCI34051 on the viability of expanded NK cells. A, VPA; B, RGFP966; C, CAY10603; D, PCI34051. The data are expressed as mean ± S.E.M. of three independent experiments. *, P<0.05; **, P<0.01; ***, <0.001.
